# COVID-19—Infections and Immunization of Inmates in Penitentiary Institutions in Poland in 2021

**DOI:** 10.3390/ijerph192113725

**Published:** 2022-10-22

**Authors:** Anna Augustynowicz, Beata Bachurska, Michał Wójcik, Mariola Borowska, Aleksandra Czerw, Janusz Opolski, Karolina Słabicka, Jarosław Pinkas

**Affiliations:** 1 Department of Economics and Medical Law, Medical University of Warsaw, 02-091 Warsaw, Poland; 2 School of Public Health, Centre of Postgraduate Medical Education of Warsaw, 01-813 Warsaw, Poland; 3 Institute of Legal Studies, College of Social Sciences, University of Rzeszów, 35-959 Rzeszów, Poland; 4 Faculty of Law and Administration, University of Silesia in Katowice, 40-007 Katowice, Poland; 5 Department of Economic and System Analyses, National Institute of Public Health—NIH—National Research Institute, 00-791 Warsaw, Poland; 6 Faculty of Engineering and Management, University of Ecology and Management in Warsaw, 00-792 Warsaw, Poland; 7 Medical Faculty, Lazarski University, 02-662 Warsaw, Poland

**Keywords:** inmates, SARS CoV-2 virus infections, quarantine, COVID-19 vaccination

## Abstract

Susceptibility to infection and the risk of a severe course of the disease caused by SARS-CoV-2 among inmates are greater than in the general population. Therefore, it is extremely important to control infections in penitentiary institutions and to vaccinate as many inmates as possible. The objectives of the study are to present the number and percentage of inmates quarantined, infected with the SARS CoV-2 virus, and vaccinated against COVID-19 in 2021, and to describe the rules and organization of immunization. The information presented in the study was obtained from the Ministry of Justice in the form of access to public information. In 2021, 2065 cases of SARS CoV-2 infection were detected among inmates, and 5707 people were quarantined. The waves of infections among inmates in Poland ran parallel to those in the general population. Immunization of inmates began at the turn of February and March 2021. It took place in accordance with the provisions of the National COVID-19 Immunization Program. The program ensured equality of the inmates’ population with the group to which individual inmates belong in the community. In 2021, nearly half of the inmates were covered by the full vaccination course. Inmates were vaccinated immediately after the vaccines were made available. There have been cases of refusals among inmates. There are no data that could determine the magnitude of the phenomenon and its exact causes.

## 1. Introduction

The COVID-19 pandemic poses great challenges to societies. Although epidemics of infectious diseases have occurred throughout history, the world was not prepared for a phenomenon that was certain to occur. Only the etiological factor could come as a surprise. If we are to secure our civilization, scientific research, enhanced health infrastructure, international collaboration, health education, biodiversity protection and ample funding will all need to be deployed [1].

It has been known for a long time that penitentiary institutions may become the epicenter of infectious diseases, because they provide ideal conditions for the transmission of pathogens. They are often overcrowded, it is difficult to ensure isolation, access to sanitary facilities is varied, and ensuring social distance is very difficult [2,3,4,5]. Penitentiary institutions are not fully isolated from the outside world. As a consequence, the SARS-CoV-2 virus can be transmitted to and from the surrounding community through the movement of personnel and visitors for different reasons [6]. In addition, non-communicable chronic diseases occur among inmates to a greater extent than in the general population. Thus, the susceptibility to infection and the risk of a severe course of the COVID-19 are higher [7,8].

Just days after the announcement of the COVID-19 pandemic, the WHO issued detailed guidelines on what should be done in penitentiary institutions to counter the epidemiological situation. The organization also warned that attempts to control the disease globally could fail if special attention is not paid to the situation in penitentiary institutions [9].

In all penitentiary systems, a number of solutions were introduced to prevent the virus from spreading. They were generally related to the improvement of sanitary conditions and the reduction of external contact [10]. The problem of reducing the number of inmates as an important factor in infection control has been discussed in particular [11,12,13]. 

In Poland, on 2 March 2020, the Director General of the Prison Service appointed a Team for Coordination of Actions Taken in Relation to Epidemiological Threat in Organizational Units of the Prison Service, which developed algorithms for dealing with suspected infection or infection of an inmate with the SARS CoV-2 virus. A decision was made to conduct an ongoing information campaign against the SARS CoV-2 virus threat directed at inmates. Additionally, it was recommended to suspend visits, external employment of inmates and religious services; it was recommended to limit transport activities and the movement of inmates on the premises of the units as much as possible; group cultural, educational, sports and therapeutic activities were limited to the necessary minimum [14].

In Poland, from 1 March 2020 to 31 December 2020, 599 cases of infection among inmates were detected. The first case of SARS CoV-2 infection was recorded on 24 March 2020. In the period from March to 30 September 2020, isolated cases of infection with the SARS CoV-2 virus were reported. The number of new infections in these months did not exceed 10 people, and ranged from 0.005% in March to 0.013% in September. Since October 2020, the number of people infected and quarantined has increased significantly. The highest number of infections (284) was observed in November, which accounted for 0.417 of the population of inmates that month. That month, 670 inmates were quarantined [14].

In addition to ensuring access to clean water, immunization is believed to be the second public health measure that can prevent premature death [15]. The development of vaccines against COVID-19 and confirmation of their effectiveness changed the prospects of fighting this disease, including in penitentiary institutions [16,17]. 

The objectives of the study are (1) to present the number and percentage of inmates quarantined and infected with SARS CoV-2 in the period from 1 January to 31 December, 2021; (2) to present the number and percentage of inmates vaccinated against COVID-19 by 31 December 2021; (3) to describe the rules and organization of the immunization of inmates.

## 2. Methods

All information presented in the Section 3 was obtained from the Ministry of Justice pursuant to the provisions of the act on access to public information [18]. Data were obtained on the numbers of inmates diagnosed with SARS CoV-2 infection, quarantined and vaccinated against COVID-19.

Those who were exposed to the disease caused by SARS-CoV-2 (COVID-19) or were in contact with a source of biological pathogens causing it were quarantined [19]. A positive RT-PCR test for SARS-CoV-2 infection was considered a laboratory confirmed case. According to the decision of the Minister of Health, from 3 November 2020, antigen tests can also be used in Poland to confirm COVID-19 in symptomatic patients. According to WHO recommendations, antigen tests are used (with a sensitivity of ≥80% and a specificity of ≥97% compared to the genetic method) in the case of the lack of availability of molecular tests or in a situation where the extended waiting time for the RT-PCR test result is an obstacle to the use of this diagnostic method in clinical practice [20]. Rapid antigen testing should be used within the first 5 days of onset of symptoms, when the viral load is highest.

Data on the number of inmates vaccinated with the first dose of the two-dose vaccine, two doses, and a single-dose vaccine by the end of 2021 were also provided, as well as data on the percentage of fully vaccinated inmates (two doses or a single-dose vaccine). The basis for calculating the percentage of vaccinated persons in individual District Inspectorates of the Prison Service was the information obtained from the Information and Statistics Bureau of the Central Board of the Prison Service in 2021. It showed that 145,558 people were held in total in penitentiary units.

Four vaccines against COVID-19 are available in Poland and are also used to vaccinate inmates: an mRNA vaccine called Comirnaty (BNT162b2), developed by Pfizer and BioNTech; an mRNA vaccine named Spikevax (mRNA 1273) developed by Moderna; an AstraZeneca vector vaccine named Vaxzevria (ChAdOx1 nCoV-19); a vector vaccine named Jcovden (COVID-19 Vaccine Janssen) (Ad26.COV.2-S). 

## 3. Results

Table 1 shows the number of cases referred to quarantine and the percentages in the individual months of 2021.

From 1 January to 31 December 2021, 2065 cases of SARS CoV-2 infection were detected among inmates, and 5707 inmates were referred to quarantine. In the period from January to April, the number of infected inmates increased. That period covers 55.01% of all infections. Since May, a decrease in the number of infected inmates has been observed. Another increase in cases was observed, starting from October 2021. The highest number of infections occurred in December 2021 (575, or 27.84% of all infections).

From 1 January to 31 March 2021, the number of people referred to quarantine increased. In April, a slight decrease in the number of people referred to quarantine was observed. In these months, 58.06% of all quarantine referrals were made. The highest number of referrals to quarantine was issued in March (1162), the lowest in September (87).

**Figure 1:**
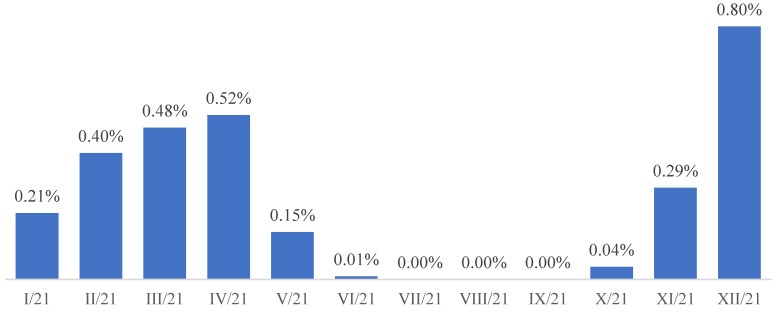
The percentages of infected inmates in individual months of 2021.

**Figure 2:**
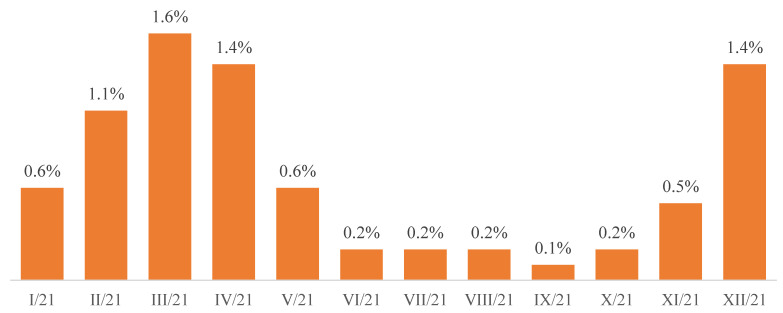
The percentages of people referred to quarantine in individual months of 2021.

The dynamics of the percentage of people infected with the SARS CoV-2 virus as well as the dynamics of the percentage of people referred to quarantine (Figure 1 and Figure 2) among inmates in the period from January to December 2021 are presented. 

Immunization against COVID-19 among inmates was preceded by an information campaign addressed directly at this group. Inmates were informed on an ongoing basis about the National COVID-19 Immunization Program [21] and the benefits of receiving the vaccine through broadcasts over radio stations, as well as during individual talks with representatives of medical professions. As of 15 February 2021, 63% of the general population of inmates in penitentiary institutions declared their willingness to be vaccinated against COVID-19.

Immunization against COVID-19 among inmates began at the turn of February and March 2021. It took place in accordance with the provisions of the National COVID-19 Immunization Program. This program defined, inter alia, the order of vaccinations by distinguishing four stages (0, I, II and III) and indicating persons authorized for immunization in particular stages. In stage 0, employees of the health care sector (medical professionals, administrative and technical employees, academic teachers at medical universities and students), employees of nursing homes and municipal social welfare centers, and personnel of sanitary and epidemiological stations were vaccinated. Stage I included residents of social welfare homes, care and treatment facilities, nursing and care facilities and other places of stationary residence, as well as people over 60 years of age in order from the oldest (with priority to those who are professionally active, are undergoing diagnostics and treatment requiring repeated or continuous contact with health care facilities and with comorbidities increasing the risk of a severe course of COVID-19), uniformed services, and teachers. Stage II provided for the immunization of people under 60 years of age with chronic diseases that increase the risk of severe COVID-19 (the list of comorbidities included chronic kidney diseases, neurological deficits, lung diseases, cancer, diabetes, COPD, cerebrovascular diseases, arterial hypertension, immunodeficiency, diseases of the cardiovascular system, chronic liver diseases, obesity, diseases related to nicotine addiction, bronchial asthma, thalassemia, cystic fibrosis, sickle cell anaemia), or under diagnostics and treatment, requiring repeated or continuous contact with health care institutions, and people directly ensuring the functioning of the basic activities of the state and at risk of infection due to frequent social contacts. Stage III provided for the immunization of entrepreneurs and employees of sectors closed due to the epidemic, and the general immunization of the rest of the adult population. The program did not explicitly refer to the immunization of inmates. Nevertheless, when analyzing its provisions, it should be recognized that it ensured equality of the population of inmates with the group to which individual inmates belong in the community. Inmates were vaccinated in the groups indicated in Stages I–III, taking into account age groups and possible comorbidities increasing the risk of a severe course of COVID-19. Each inmate was offered a vaccine. The immunization of inmates, as well as of the entire population, was voluntary. Inmates could decide whether to receive the COVID-19 vaccine. They may also have refused to be vaccinated with a specific vaccine. As such, they were waiting for the availability of a vaccine they accepted. 

Initially, inmates were vaccinated outside penitentiary institutions, at immunization points. In May 2020, the Prison Service received permission to carry out immunization against COVID-19 on its own. As of 7 June 2021, vaccines were administered by prison medical personnel. At the same time, immunization was also performed outside—depending on the availability of vaccines.

Information on the numbers and percentages of inmates vaccinated in penitentiary institutions in the full vaccination schedule (two doses or a single-dose product) and the number of people vaccinated with only one dose is presented in Table 2.

In 2021, nearly half (49.2%) of the inmates were covered by the full vaccination course. The percentage of inmates vaccinated in individual Inspectorates does not vary greatly. The highest percentage of vaccinated inmates was observed in the Inspectorates of Wrocław, Katowice, Bydgoszcz and Warsaw.

## 4. Discussion

In Poland, the third and fourth waves of SARS-CoV-2 infections were recorded in 2021. The beginning of the third wave was in mid-February 2021, peaking on April 1 with 35,251 new cases of SARS-CoV-2—the highest daily number of infections since the beginning of the pandemic in Poland. In April 2021, in the third wave, the number of infections among inmates was the highest. From September 2021, the number of infections among inmates began increasing. The peak of the fourth wave was in December 2021. On December 1, the highest daily number of infections in this wave was recorded—29,064 new cases. In December the highest number of infections was observed among inmates in 2021—575 [22]. The data clearly show that the waves of infections among inmates in Poland ran parallel to the waves of those in the general population—with the increase in the number of infections in the general population, the number of infections among inmates increased.

When comparing the population of inmates in Poland to the general population, higher percentages of infection with the SARS CoV-2 virus were not recorded. In other countries, the distribution of infections seems to be different. In the USA, the case rate was 5.5 times higher than that of the US general population, escalating much more rapidly in penitentiary institutions [23]. N. Marquez et al. examined COVID-19 and death rates during the first 52 weeks of the pandemic, e.g., from 5 April 2020 to 3 April 2021. The inmate population weekly incidence rate peaked during the month of December, and has since declined, but the cumulative toll of COVID-19 has been several times greater among the inmate population than the US general population [24]. MF. Aebi and M. Tiago analyzed trends in the European inmate population from 1 January to 15 April 2020. They observed a decreasing trend, probably due to three factors: a reduction in crime, a decrease in the activity of the justice system and the release of inmates as a preventive measure [25]. In Italy, S. Mazzili et al. assessed the extent and dynamics of COVID-19. During both epidemic waves, inmates and personnel had a higher risk of COVID-19, and incidence rates were higher amongst inmates [26]. In late July 2020, Latin America and the Caribbean led the world in total cases of COVID-19, and incidence rates among inmates were higher compared to the general population. In Brazil, for example, there were 184 cases per 10,000 residents, as compared to 389 among inmates [27].

The immunization of inmates in Poland was carried out in accordance with the provisions of the National COVID-19 Immunization Program. Although there was no explicit mention of the immunization of inmates, they were treated equally with other citizens. In many countries, national programs were the basis for activities in this area. However, certain differences were observed. In terms of the approach to immunization of inmates in penitentiary institutions, four groups of countries can be distinguished: countries that have explicitly prioritized prisons, including prison populations as a higher-risk group; countries that have included prisons within plans or roll-out, but not as a (high) priority group; countries that ensure the equivalence of prison populations or personnel with the group that the individuals would fall within in the community; and countries that have not specifically referred to prisons, prison populations, or staff at all in national immunization programs [28]. Inmates in penitentiary institutions have been identified as a priority group in terms of access to immunization against COVID-19, e.g., in Portugal and Austria. Countries with national immunization plans that include inmates, but not as a priority group, include Latvia, Greece, Spain, Ukraine, Canada, Mexico, Honduras, Paraguay, Argentina, Chile, Angola, and Namibia. The third group of countries includes, for example, Romania, Bulgaria, Colombia, Peru, Ecuador and Brazil. The fourth approach is represented, inter alia, by Poland, as well as Sweden, Finland, Estonia, Czech Republic, Russia, Turkey, Nigeria, USA, Bolivia, Uruguay, Thailand, Cambodia, and Malaysia [29].

In Poland, the immunization of inmates against COVID-19 began relatively early, at the turn of February and March 2021. We have observed a large variation in terms of the starting of immunizing inmates. Some countries did not distribute a single vaccine to inmates before June 2021, while others reported that immunization started as early as late March. For example, in France, the immunization of inmates began in January 2021, in Portugal, Morocco and Malta in March, in Thailand in May, in the Philippines in June, and in Iran in August of 2021 [28]. 

In Poland, in 2021, nearly half (49.2%) of all inmates were fully vaccinated, although there are also examples of countries that have vaccinated a much higher percentage of inmates. In Malta, the rate is 94.2%, in Italy and Switzerland, slightly over 80%, and in Kazakhstan and Chile it is 80%. However, there are also countries with much smaller percentages of vaccinated inmates, at less than 10% [28]. 

Inmates refusing to get vaccinated was observed. There are no studies that have allowed us to assess the magnitude of the phenomenon and the exact causes. Individual studies indicate that the most common cause of refusals is fear of side effects [30,31].

## 5. Conclusions

In the Polish penitentiary system, inmates were vaccinated immediately after the vaccines were available.

The waves of COVID-19 cases among inmates and the general population ran parallel.

The basis for the immunization campaign was the National COVID-19 Immunization Programme. This program did not assume any particular preferences for the population of inmates.

In 2021, 49.2% of inmates were fully vaccinated. 

There have been cases of refusals among inmates. No data could determine the magnitude of the phenomenon and its exact causes.

## Figures and Tables

**Table 1:** Number of infected prisoners referred to quarantine.

Month	Inmates				
	Infected		Referred to Quarantine		Total Number of Inmates As of the Last Day of the Month
	n	%	n	%	
January	142	0.21%	425	0.60%	68,852
February	281	0.40%	742	1.10%	70,117
March	339	0.48%	1162	1.60%	71,297
April	374	0.52%	985	1.40%	71,258
May	105	0.15%	441	0.60%	71,375
June	9	0.01%	157	0.20%	71,640
July	0	0.00%	113	0.20%	71,960
August	2	0.00%	115	0.20%	71,907
September	0	0.00%	87	0.10%	71,291
October	29	0.04%	119	0.20%	71,391
November	209	0.29%	342	0.50%	71,546
December	575	0.80%	1019	1.40%	71,874

**Table 2:** Number of people vaccinated in penitentiary institutions in Poland as of 31 December 2021.

District Inspectorate of the Prison Service	Number of Inmates Vaccinated with One Dose	Number of Inmates Vaccinated with Two Doses	Number of Inmates Vaccinated with a Single-Dose Product	% Inmates Fully Vaccinated
Białystok	22	159	3626	2.6%
Bydgoszcz	38	371	6153	4.5%
Gdańsk	162	422	4378	3.3%
Katowice	726	841	5817	4.6%
Koszalin	832	463	2493	2.0%
Kraków	20	474	4171	3.2%
Lublin	14	285	3725	2.8%
Łódź	1233	1114	3362	3.1%
Olsztyn	779	752	3030	2.6%
Opole	106	1123	2903	2.8%
Poznań	320	612	4957	3.8%
Rzeszów	2105	1947	1384	2.3%
Szczecin	180	238	3749	2.7%
Warszawa	308	282	6015	4.3%
Wrocław	371	441	6296	4.6%
TOTAL	7216	9524	62,059	49.2%

## Data Availability

All data are available from the corresponding author.
